# Structure Formation in Engineered Wood Using Wood Waste and Biopolyurethane

**DOI:** 10.3390/ma17164087

**Published:** 2024-08-17

**Authors:** Aurelija Rimkienė, Agnė Kairytė, Sigitas Vėjelis, Arūnas Kremensas, Saulius Vaitkus, Jurga Šeputytė-Jucikė

**Affiliations:** Building Materials Institute, Faculty of Civil Engineering, Vilnius Gediminas Technical University, Linkmenu str. 28, LT-08217 Vilnius, Lithuania; aurelija.rimkiene@vilniustech.lt (A.R.); sigitas.vejelis@vilniustech.lt (S.V.); arunas.kremensas@vilniustech.lt (A.K.); saulius.vaitkus@vilniustech.lt (S.V.); jurga.seputyte-jucike@vilniustech.lt (J.Š.-J.)

**Keywords:** wood waste, bulk density, granulometric composition, engineered wood, compressive stress, tensile strength, microstructure

## Abstract

This research aims to find suitable processing methods that allow the reuse of wood waste to produce wood waste-based engineered wood logs for construction that meet the strength requirements for structural timber for sawn structural softwood. Three types of wood waste were examined: wood packaging waste (W), waste from the construction and furniture industry (PLY), and door manufacturing waste (DW). The wood waste was additionally crushed and sieved, and the granulometric composition and shape of the particles were evaluated. The microstructure of the surface of the wood waste particles was also analysed. A three-component biopolyurethane adhesive was used to bind wood waste particles. An analysis of the contact zones between the particles and biopolyurethane was performed, and the adhesion efficiency of their surfaces was evaluated. Analysis was performed using tensile tests, and the formation of contact zones was analysed with a scanning electron microscope. The wood particles were chemically treated with sodium carbonate, calcium hypochlorite, and peroxide to increase the efficiency of the contact zones between the particles and the biopolyurethane adhesive. Chemical treatment made fillers up to 30% lighter and changed the tensile strength depending on the solution used. The tensile strength of engineered wood prepared from W and treated with sodium carbonate increased from 8331 to 12,702 kPa compared to untreated waste. Additionally, the compressive strength of engineered wood made of untreated and treated wood waste particles was determined to evaluate the influence of the wood particles on the strength characteristics.

## 1. Introduction

Wood waste is the leftover wood products from several industries, including agriculture, railroad construction, wood packaging, demolition, construction, and wood processing [1,2,3,4,5]. Recycling and reusing between 80 and 90% of waste from building and demolition projects is possible, provided they are separated and used appropriately [6]. Large quantities of medium- and high-density fibreboard waste are received from the furniture sector. Such waste is used to produce other composites burnt or landfilled after their end of life [7,8,9,10]. Wood waste can be divided into three classes: treated materials, untreated or natural materials, and materials that should be disposed of because they cannot be reused [11,12,13]. According to European directives, wood waste can only be processed or used as an energy source if it meets public safety and environmental standards [12,14]. For this reason, all wood waste must be carefully sorted. One of the most effective sorting methods is near-infrared spectroscopy (NIRS), which is widely used in other processing steps due to its speed, affordability, ease of use, and possible direct classification in the production line [12,14,15,16].

Wood waste used to produce new products can be processed mechanically, thermally, and chemically [17,18,19,20,21]. Additional mechanical treatment helps to improve the adherence properties between the wood particles and the binding material, allows the desired fraction to be obtained, and helps remove metal inclusions with magnets [22,23]. Chemical treatment improves the adhesion between the binding material and filler and impacts absorbency; hydrophobicity; microbiological, heat, fire, and mechanical resistances; and other properties [23,24,25]. Several groups of scientists have investigated the influence of the thermal process on the efficacy of removing soluble substances and hazardous components from wood waste at certain temperatures [23,26]. Synthetic resins made from non-renewable petrochemicals are still commonly used as wood adhesives in the furniture, architecture, automotive, and packaging sectors. Formaldehyde accounts for more than 95% of all adhesives used to produce wood-based panels. Such resins are synthesized by reacting formaldehyde with various substances, including urea, phenol, melamine, resorcinol, or combinations [27,28,29,30,31]. There are two methods for extracting urea–formaldehyde from wood waste: condensation and hydrolysis and low-temperature pyrolysis.

Steam cooking under a pressurized saturated steam atmosphere can separate the urea–formaldehyde resin from the wood fibres in wood waste. It was discovered that 80 °C for two hours in the presence of oxalic acid was the ideal temperature to remove the urea–formaldehyde resin from medium-density fibreboards [26,32,33,34]. Due to less corrosive ingredients and the lack of a separation stage, pure steam treatments are less expensive than those under acidic conditions and are more environmentally friendly. Another study also demonstrated that treatment with water vapour at higher temperatures could promote the hydrolysis reaction. The water steam at 150–190 °C for 10–20 min. eliminated approximately 80% of the resins, as stated in the research [26]. Notably, up to 60% wood waste can be used as a fuel source, aggregate for concrete composites and wood–plastic composites, and raw material for the production of chipboards and fibreboards and obtain stabilizing binders, methanol, turpentine, acetone, and acetic acid [11,19,35,36,37,38,39].

One type of wood composite is engineered wood. As stated in the literature [40], engineered wood or artificial wood can be defined as a composite material made by bonding or reinforcing wood strands, particles, fibres, or wood veneers with an adhesive to form a composite material. Unlike solid wood, engineered wood has excellent structural stability. It is often designed to overcome the disadvantages of natural wood, such as warping, shrinkage and swelling due to changes in humidity and temperature. Engineered wood has been engineered to offer superior strength, durability and dimensional stability, making it an ideal alternative for various construction and manufacturing applications [41,42,43].

Fibrous and particulate wood are the two main categories into which wood composites are often divided. In industrial applications, lignocellulosic materials can be applied as reinforcements or fillers for thermoplastic polymers. Small fibres with high aspect ratios in wood plastic composites are advised to be utilized because they are more homogeneously dispersed throughout the matrix compared to long fibres and have a greater specific surface area, which enhances compatibility and works as reinforcement. Wood particles can be any shape and have almost equal dimensions in all directions; they are primarily used as fillers [44]. Instead of being landfilled or burned, wood waste fibres, made from residues from producing wood panels for furniture, can be utilized as a substitute for cellulose fibres in stone mastic asphalt mixtures as stabilizing binders [45]. With various uses in building construction, recycled wood-based materials, such as pallets and construction waste bonded with the cementitious matrix, offer the construction industry a viable alternative. Since wood cement composites are environmentally friendly and practical to produce, they are an excellent option for use in green buildings [46,47,48]. Low-cost and high-strength wood fibre/polypropylene composites can be produced with 5–55 wt.% recycled wood fibre from the waste sawdust reinforced with a polymer matrix [49,50,51,52]. However, more than 50% of wood particles tend to decrease mechanical resistance.

Because of different chemical structures, bio-based binding materials are frequently classified into several groups, such as lignin, tannin, plant and animal proteins, and carbohydrates. A soybean-based adhesive designed for indoor plywood is the only wholly biomass-based wood adhesive available on the market. For the manufacturing of biomass-based adhesives, carbohydrate-based materials, such as cellulose, sucrose, chitosan, starch, glucose, natural rubber, carboxylic acid, and vegetable-based oils, are essential raw materials. According to some published research, the best action is to develop renewable and environmentally friendly wood adhesive by replacing formaldehyde and urea with two raw biomass materials such as cellulose and sucrose. It will also significantly improve the bonding performance of the adhesive [53,54,55,56,57].

The most usual types of engineered wood include multi-layer hardwood products (made from beech, birch, and mahogany), multi-layer softwood products (made from cedar wood, fir, and pine), tropical multi-layer products, decorative multi-layer products, particle-based products, medium-density fibre, and high-density fibre products [40]. The most commonly used type of engineered wood for the construction industry is wood particle-based products. Compared to other building materials, this type of engineered wood shows the following qualities: it is lightweight and mechanically robust; its thermal conductivity is low; it effectively dampens noise; it is resistant to the impact of harmful chemicals; it positively affects the microclimate of rooms by absorbing moisture in higher humidity conditions and releasing it in lower ones; it is resilient to the influence of harmful biological factors; and finally, it is made of renewable material [57,58,59,60,61]. However, wood-based particle products have drawbacks such as an insufficient strength, load, and pressure-bearing capacity when used for structural purposes [40]. Therefore, it is important to overcome these negative aspects and develop more robust composite materials, such as wood particle-based engineered wood products, that could mimic the properties of a natural wood. Even though wood particle-based engineered wood products do not have a harmonized standard and there is no strict definition, classification, or specific requirement for the indicators of the created products, it is usually subject to the condition that the properties of the engineered products are similar or better than products made from natural wood. Therefore, to use the obtained wood particle-based engineered wood products as an alternative to natural wood for structural purpose, their compressive strength must vary from 4.3 to 6.3 MPa across the fibres and from 16 to 26 MPa along the fibres. Meanwhile, the tensile strength of wood across the fibres is low, only 0.3–0.4 MPa, and along the fibres, it ranges from 8 to 24 MPa [62]. The main engineering tasks that enable the production of engineered wood have remained almost unchanged for several decades: selection of the binder and its amount, adjustment of the coarseness and moisture content of the wood filler, selection of the appropriate pressing level, curing temperature and duration. With the development of the binder industry, it became possible to use more environmentally friendly binders that do not require thermal treatment. Polyurethane-based binders are usually used without thermal treatment. The essence of the binding of these binders is the reaction of two chemical elements. Such binder hardening allows the production of products of unlimited dimensions, which means a broader range of possible products.

This work aims to prepare and analyse different wood wastes and compare the properties of wood waste-based engineered wood logs intended for the production of sawn construction wood substitutes such as construction logs, special assortment blocks, and other sawn wood construction products which meet the strength requirements of EN 338 [63]. The main tasks of the work are to check whether the used wood waste is suitable for the production of engineered wood, whether biopolyurethane can be used as a binding material for binding this waste, and how strong contact zones are formed without thermal treatment. In this work, the granulometric composition and bulk density of the wood waste, the effect of chemical treatment on the surface of the wood waste, and the formation of contact zones between the wood waste particles and the biopolyurethane adhesive were evaluated. Tensile tests evaluated the effectiveness of contact zone formation, while compression tests evaluated the effect of wood waste particles on the strength of engineered wood.

## 2. Materials and Methods

### 2.1. Raw Materials

Three types of wood waste were used as fillers in the production of engineered wood: wood packaging waste (W), construction demolition and furniture waste (PLY), and door manufacturing waste (DW). W and PLY were taken from the waste storage and processing site of UAB Ekobazė (Žariju st. 2, Vilnius, Lithuania). In contrast, DW was taken from the manufacturing waste site of AS Viljandi Windows & Doors (Puidu 6, Viljandi, Estonia).

A biopolyurethane was used as a binding material to prepare engineered wood. The main advantages of biopolyurethane binder are that the composites with biopolyurethane do not require additional heat treatment and the majority of fossil resources are replaced by products of plant origin. The ratio of wood waste to biopolyurethane binder was 50/50. The biopolyurethane binder consisted of isocyanate, polyol, and natural rapeseed oil. Their percentages in the composition of a binding material were 35, 44, and 21%, respectively. Lupranat M20S polymeric 4,4-diphenylmethane diisocyanate with 31.5% NCO (BASF, Berlin, Germany) was used as a hardener. Polyol Biopolyol RD (SIA PolyLabs, Riga, Latvia) was used for research. Rapeseed oil-based polyol had a hydroxyl number of 350 KOH/g and less than 0.2% water. Rapeseed oil was produced by Lomista UAB (Kaišiadorys, Lithuania).

### 2.2. Preparation of Wood Waste

All the obtained waste was also mechanically crushed, sieved, and chemically treated, and the granulometric composition and microstructure of the surfaces of the particles were also evaluated. Wood waste, except for DW, was further crushed with a hammer mill (SC Radviliškio mašinų gamykla, Radviliškis, Lietuva). The obtained DW particles were fine in size and passed through a 20 mm sieve mesh. Additionally, all three types of wood waste were sieved with a sieve column of 20, 10, 5, 2.5, 1.25, 0.63, 0.315, and 0.1 mm sieves (Glenammer Sieve Ltd., Ayr, Scotland).

### 2.3. Modification of Wood Waste

Comparisons were made between untreated and sodium carbonate (S)-, calcium hypochlorite (C)-, and peroxide (P)-treated W, PLY, and DW particles. Chemical treatment agents were selected according to two criteria: they significantly affect soluble substances contained in wood or its composites and cause the least damage to human health during the application. The concentration of selected agents was 10% based on the weight of the wood waste. Each solution was poured into a metal pot containing wood waste particles, covered with water, and boiled for 1 h. Then, it was set to cool for 23 h. The excess solution in wood waste was drained through a metal sieve during the cooling. Then, the pot was filled with water again. These steps were repeated six times in a row. After that, the wood waste particles were placed on a metal sieve and left to drain for 24 h. Further, the particles were placed in a drying chamber and dried to a constant mass.

### 2.4. Preparation of Wood Waste-Based Engineered Wood Logs

First, polyol and rapeseed oil were mixed for a minute at 1800 rpm. After that, the intended amount of isocyanate is added to the mixture produced and stirred for another 10 s. The prepared biopolyurethane binder was then poured onto the weighted wood waste particles and thoroughly mixed for a minute at 1800 rpm. The mixture was poured roughly into moulds, pressed with a load of 1.5 MPa using a pneumatic–hydraulic press Tongrun T40 (Shanghai Tongrun Imp. & Exp. Co., Ltd., Shanghai, China) and kept compressed for 24 h before demoulding and cutting the wood waste-based engineered wood logs (further in the text, engineered wood) to the required dimensions.

Before crushing and evaluating granulometric composition and bulk density, all wood waste was conditioned for at least 72 h in 50 ± 5% relative air humidity and 23 ± 2 °C temperature.

### 2.5. Testing Methods

Before crushing W, PLY, and DW, an evaluation of granulometric composition and bulk density was implemented. All wood waste was conditioned for at least 72 h in 50 ± 5% relative air humidity and 23 ± 2 °C temperature.

Tensile strength tests were performed according to the requirements of the EN 1607 standard [64]. Five specimens were used for each type of engineered wood. Before the test, the specimens were conditioned for 24 h at a temperature of (23 ± 2) °C and a humidity of (50 ± 5)%.

Compressive strength tests were performed according to the requirements of the EN ISO 29469 standard [65]. Five specimens were used for each type of engineered wood. Before the test, the specimens were conditioned for 24 h in an environment with a temperature of (23 ± 2) °C and a humidity of (50 ± 5)%.

A scanning electron microscope (SEM), the EVO-50 (Carl Zeiss SMT, Oberkochen, Germany, 2006), was used for research studies of the structure of wood waste particles and contact zones between particles and binding material.

## 3. Results and Discussion

### 3.1. Parameters of Wood Waste

**Figure 1:**
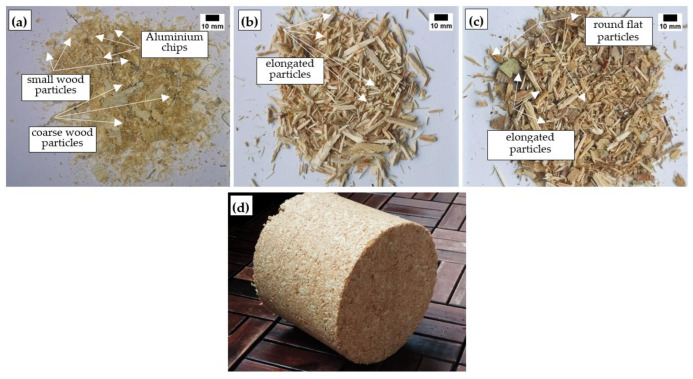
Prepared wood waste from different industries: (**a**) DW; (**b**) W; (**c**) PLY; and (**d**) DW-based engineered wood log.

**Figure 2:**
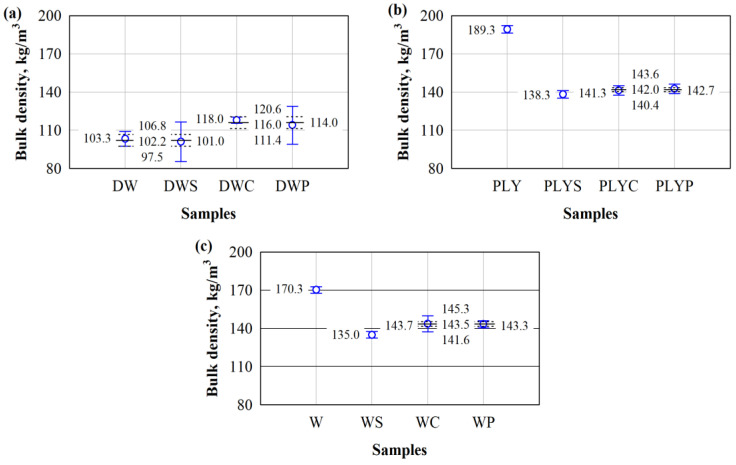
Bulk density of wood waste particles from different industries: (**a**) DW; (**b**) PLY; and (**c**) W; untreated or treated with S, C, or P; ○—mean value of the sample; -----—sample limits; ^__^—upper, lower, and mean values of identical samples.

Samples of wood waste used for the tests are shown in Figure 1. Figure 1a shows that fine particles dominate in the DW and that the particles are circular. In addition, elongated aluminium chips are visible. Aluminium chips are formed during the production of wood-aluminium door frames and leaves when they are processed to exact dimensions. Elongated particles of different diameters characterize the W (Figure 2b). In the PLY (Figure 1c), round particles of various sizes and elongated particles of various diameters are dominated. Additionally, Figure 1d presents the DW-based engineered wood log.

Figure 2 presents the density distribution of untreated and chemically treated wood waste particles.

All untreated wood waste particles have different densities. In addition, the selected chemical treatment of wood waste particles not only impacts the density, but the effect is very different. DW has the lowest density among chemically untreated wood waste particles (Figure 2a). As shown in Figure 1, the DW contains aluminium chips. The aluminium chips are thin, long, and needle-shaped, but they are hard enough to form a specific framework throughout the waste volume and support the weight of the wood particles. In this way, the aluminium chips prevent the wood particles from compacting, especially the larger ones, and result in a lower bulk density value. In Figure 2, it can be seen that chemical treatment affects the bulk density of all wood waste particles. First, it was observed that the lowest bulk density for all wood waste particles is obtained with S and the highest one with C treatment. During chemical treatment, fine particles and various soluble substances are washed out by the washing procedure. This scenario corresponds to the treatment of wood waste particles with S, where a lower density is obtained than before chemical treatment. When DW is processed with C and P, a higher density is obtained than before chemical treatment.

Because only small and soluble wood particles are washed out during chemical treatment, aluminium particles, which have a several times higher density, DW particles treated with C and P become denser due to the washed-out small wood particles, leading to an overall increase in density. The density of samples washed with S is lower because it is likely to wash soluble substances better from the walls of the wood waste particles, thus allowing the same volume of wood waste particles to remain. It is confirmed by bulk density studies with wood waste particles from PLY and W, where similar trends remain.

Statistical analysis shows that the mean values of untreated and S-treated DW densities did not differ. The statistic of the F criterion was 0.37, *p* > 0.58. This indicates a statistically insignificant difference in the results of the subject. Consequently, the coefficient of determination R^2^ = (0.084) and the corrected coefficient of determination R^2^ = (−0.14) are obtained. The mean values of the filler densities also do not differ after the respective treatment of the DW with C and P. The statistic of the F criterion is 1.30, *p* > 0.32. This indicates a statistically insignificant difference in the results of the subject. Consequently, the coefficient of determination R^2^ = (0.24) and the corrected coefficient of determination R^2^ = (0.056) are obtained. The average density of the untreated and S-treated DW will be 102.2 kg/m^3^, while for C and P-treated DW, it will be 116.0 kg/m^3^. Consequently, the difference is 1.14 times.

In the study of untreated PLY, the highest bulk density is 189.3 kg/m^3^, while the lowest, i.e., 138.3 kg/m^3^, is obtained after treatment with S (see Figure 2b). The difference between these waste particles was 1.37 times. After treating PLY with C and P, the average values of the densities of these waste particles do not differ. The statistic for the F-criterion is 1.14, *p* > 0.35. It indicates a statistically insignificant difference in the results of the subject. Accordingly, the coefficient of determination R^2^ = (0.22) and the corrected coefficient of determination R^2^ = (0.028) are obtained. After processing PLY with C and P, the average value of the densities of these waste particles will be 142.0 kg/m^3^.

The same trend is observed in the study of W particles. The highest bulk density of untreated W was 170.3 kg/m^3^, and the lowest was 135.0 kg/m^3^, obtained after the W treatment with S (see Figure 2b). The difference between these wood particles is 1.26 times. After treating W with C and P, the average values of the bulk densities of these two wood waste particles do not differ. The statistic for the F-criterion is 0.43, *p* > 0.85. This indicates a statistically insignificant difference in the results of the subject. Consequently, the coefficient of determination R^2^ = (0.011) and the corrected coefficient of determination R^2^ = (−0.24) are obtained. After treating W with C and P, the average value of the densities of these two wood waste particles will be 143.5 kg/m^3^.

Table 1 shows the results of the granulometric composition of all wood waste particles used for the study. The analysis of the results shows that particles of two sizes predominate in DW, ˃ 0.315÷1.25 and ˃ 2.5÷5. Meanwhile, in PLY and W, one size of particles predominates ˃ 2.55÷ 5 mm. Furthermore, in DW, when 0÷ 2.5 mm particles are analysed, an increase in fine particles is observed after chemical treatment compared to untreated DW particles. Fine wood particles are likely to wash out more easily, so fine aluminium particles, which are heavier, have a greater influence on the particle mass distribution.

### 3.2. Strength Parameters of Engineered Wood 

**Figure 3:**
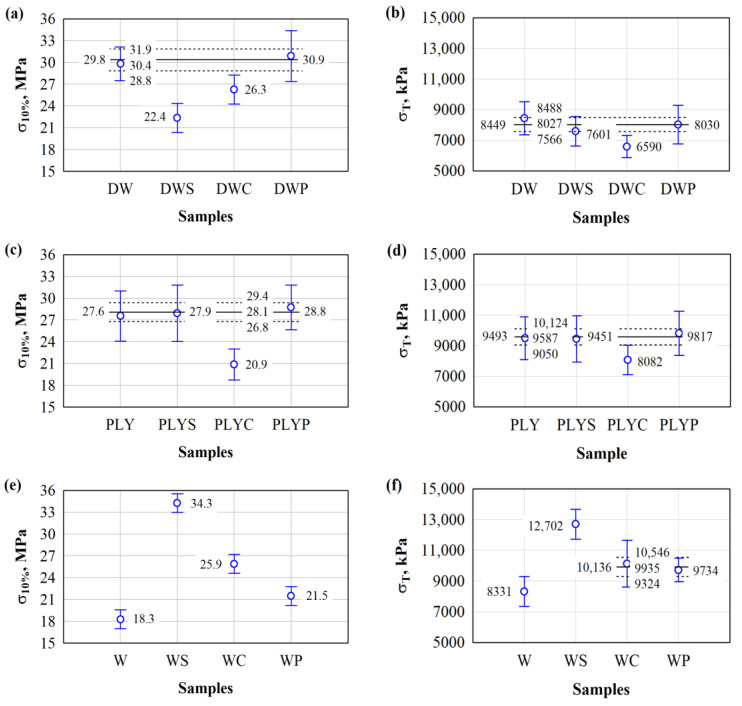
Strength parameters of engineered wood prepared from wood waste from different industries: graphs (**a**,**c**,**e**) show compressive strength results; graphs (**b**,**d**,**f**) show tensile strength results; and the marking of the specimens and symbols is the same as in Figure 2.

Next, samples were prepared from untreated and chemically treated different wood waste to assess the strength properties. Figure 3 shows the results of compressive strength (Figure 3a,c,e) and tensile strength parallel to the surfaces (Figure 3b,d,f).

The analysis of the results of the compression and tension tests show (see Figure 3) how compressive and tensile strengths are affected by various wood waste particles treatments. In Figure 3a, it can be seen that untreated DW and P-treated DW have the greatest impact on compressive strength. Analysis of variance revealed that there is no difference between the mean compressive strength values for untreated and P-treated DW composites.

The statistic for the F-criterion is 0.63, *p* > 0.46. This indicates a statistically insignificant difference in the results of the subject. Accordingly, the coefficient of determination R^2^ = (0.095) and the corrected coefficient of determination R^2^ = (−0.056) are obtained. Thus, the average value of the compressive strength of the untreated DW and P-treated DW will be 30.4 kPa. Treatment of DW with S and C results in lower compressive strength. Comparing untreated DW and P-treated DW specimens with S-treated DW specimens, the difference is 1.4 times. Meanwhile, after C treatment, this difference is 1.2 times.

In Figure 3c, it can be seen that compressive strength is mostly affected by untreated S and P-treated PLY composites. Analysis of variance reveals that there are no differences in mean compressive strength values for untreated PLY composites, S-treated PLY, and P-treated W composites.

The statistic for the F-criterion is 0.31, *p* > 0.74. This indicates a statistically insignificant difference in the results of the subject. Consequently, the coefficient of determination R^2^ = (0.064) and the corrected coefficient of determination R^2^ = (−0.14) are obtained. Therefore, the average compressive strength value of untreated PLY, S-treated PLY, and P-treated PLY composites will be 28.1 kPa. Treatment of PLY with C results in a lower compressive strength, i.e., 20.9 kPa. When comparing untreated PLY, S-treated, and P-treated PLY composites with C-treated PLY composites, the difference is 1.34 times.

After carrying out the dispersion analysis, it has been found (Figure 3e) that the mean values of the compressive strength of untreated W, S, C, and P-treated W composites are different. The statistic for the F-criterion is 17.5, *p* > 0.0. This indicates a statistically significant difference in the results of the subject. Consequently, the coefficient of determination R^2^ = (0.81) and the corrected coefficient of determination R^2^ = (0.77) are obtained. S-treated W particles have the greatest influence on compressive strength, i.e., 34.3 kPa, while the value of untreated W composites is lower, i.e., 18.3 kPa. When comparing the average compressive strength values of the S-treated W composites, the difference is ~1.87 times.

It can be seen that after using DW, PLY, or W as composite material aggregates and treating them accordingly, the highest compressive strength is obtained after treating the W particles with S.

The results of engineered wood tensile tests show (Figure 3b) how the untreated and treated DW influence the tensile strength of the composites. It is found that the average values of the tensile strength of composites with untreated, S- and P-treated DW particles do not differ. The statistic for the F-criterion is 1.48, *p* > 0.28. This indicates a statistically insignificant difference in the results of the subject. Consequently, the coefficient of determination R^2^ = (0.25) and the corrected coefficient of determination R^2^ = (0.081) are obtained. Therefore, the average tensile strength of composites with untreated DW and S- and P-treated DW particles will be 8027 kPa. C treatment of DW results in a lower tensile strength of the composites. When comparing composites prepared from untreated DW and S- and P-treated DW particles with C-treated DW particles, the difference in tensile strength is 1.2 times.

After using composites prepared from untreated PLY and S- and P-treated PLY particles, the average tensile strength values do not differ (Figure 3d). The statistic for the F-criterion is 0.19, *p* > 0.83. This indicates a statistically insignificant difference in the results of the subject. Consequently, the coefficient of determination R^2^ = (0.041) and the corrected coefficient of determination R^2^ = (−0.17) are obtained. Thus, the average tensile strength of composites prepared from the untreated DW and S- and P-treated DW particles will be 9587 kPa. PLY particle treatment with C results in lower tensile strength of the composites. The difference between the composites prepared from untreated PLY and S- and P-treated PLY particles and C-treated PLY particles is 1.2 times.

Meanwhile, after using W as an aggregate for the production of engineered wood, it is found that the specimens made of S-treated W particles have the highest tensile strength, and the specimens made of untreated W particles have the lowest tensile strength (Figure 3f). The difference between the tensile strength of these specimens is 1.5 times. After treating W with C and P, it was found that the mean tensile strength values of the specimens did not differ. The statistic for the F-criterion is 0.57, *p* > 0.48. This indicates a statistically insignificant difference in the results of the subject. Accordingly, the coefficient of determination R^2^ = (0.086) and the corrected coefficient of determination R^2^ = (−0.066) are obtained. After treating W with C and P, the average value of the tensile strength of the specimens will be 9935 kPa.

The analysis of the experimental data show that, after using DW, PLY, or W particles as an engineered wood aggregate and after treating them accordingly, the highest tensile strength is obtained for specimens prepared from S-treated W particles.

Comparing the values of compressive strength and tensile strength obtained in our work with sawn construction softwood, we see that the values of compressive strength and tensile strength of engineered wood are significantly higher than the values of compressive strength and tensile strength of softwood across the fibers, and are of similar order to the compressive strength and tensile strength of softwood values along the fibers. According to EN 338 Table 2 in [63], all the developed sawn construction softwood independently of the chemical treatment used fall into the T11–T30 classes based on the compressive strength results (compressive strength varies from 18 MPa to 30 MPa) and into the T8–T12 classes based on the tensile strength results (tensile strength varies from 8 MPa to 12 MPa) if classified separately. However, if classified according to both parameters, the T11–T12 classes could be applied.

The current study used different wood waste particles of 0–20 mm fraction. Other researchers have found that particle size significantly influences the strength characteristics of wood composites [66,67,68]. The researchers found that wood particles of 12–18 mm can achieve the highest bending strength. In another work [66], the authors obtained similar results when using wood particles with an average length of 12.2 mm, thus obtaining the highest tensile and bending strength. When wood particles with a length of 20.2 mm are used, the tensile and bending strengths decrease. The researchers also obtained lower tensile and bending strength values due to the application of 1.9 and 3.9 mm sized wood particles.

Wood must be treated to reduce the polarity gap between the wood particles and the polymer matrix. Various methods are used to treat wood particles and wood waste to improve adhesion to the binder [69,70,71]. The researchers found that the tensile strength and flexural strength increased by 29 and 27%, respectively, after wood particle treatment. In this case, compressive strength and tensile strength increased by 87 and 52%, respectively, using W particles.

### 3.3. Structure of Wood Waste

**Figure 4:**
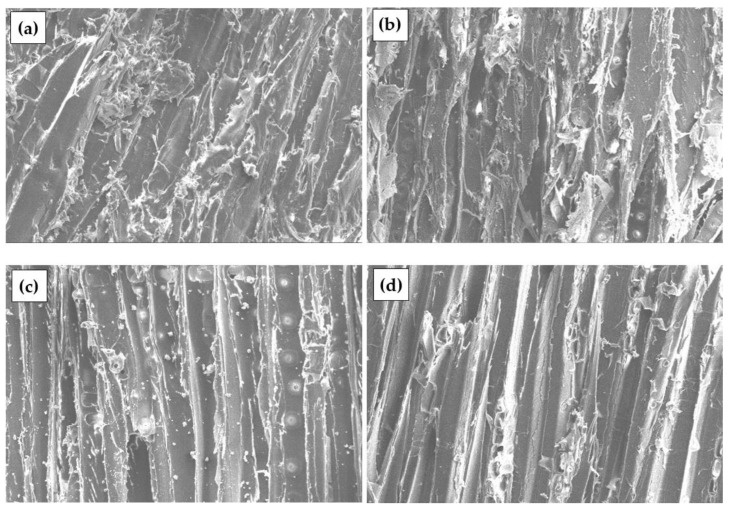
DW particles with different surface preparation, ×500: (**a**) untreated; (**b**) S-treated; (**c**) C-treated; (**d**) P-treated.

Figure 4 shows the surface of the specimens prepared from the untreated and treated DW with different chemical solutions. In chemically treated DW, dust reduction in all specimens is detected. In chemically treated DW, a reduction in dust in all specimens is detected. In the S-treated DW, a rougher sample surface and the formation of fine crystals on the sample surface caused by incompletely leached sodium carbonate are observed (Figure 4b). C-treated DW particles result in a smooth surface and large crystals due to incomplete removal of C (Figure 4c). A smooth surface without crystal formation and loss of individual layers in individual zones is observed in P-treated W particles (Figure 4d).

**Figure 5:**
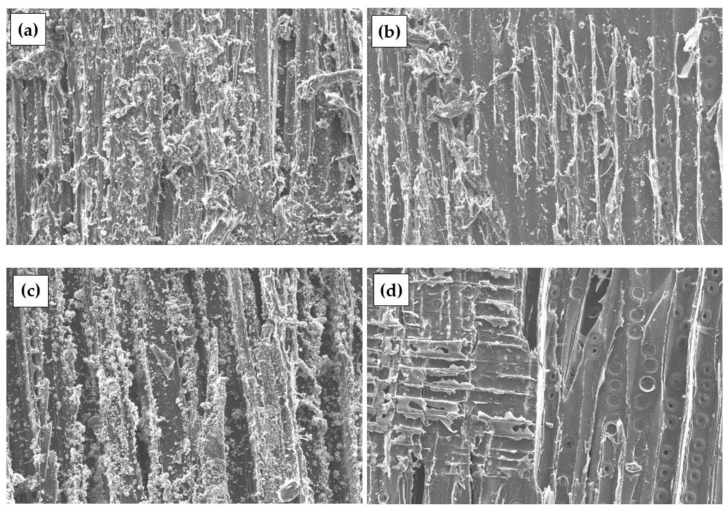
W particles with different surface preparation, ×500: (**a**) untreated; (**b**) S-treated; (**c**) C-treated; (**d**) P-treated.

Figure 5 illustrates the effects of chemical treatment on W particles, which are similar to those observed in DW particles. After the chemical treatment, a reduction in dust is noted in all samples, and the overall effect of the chemical treatment is consistent. However, a higher amount of crystal formation is observed, indicating a specific variation in the response of W particles to the chemical treatment.

**Figure 6:**
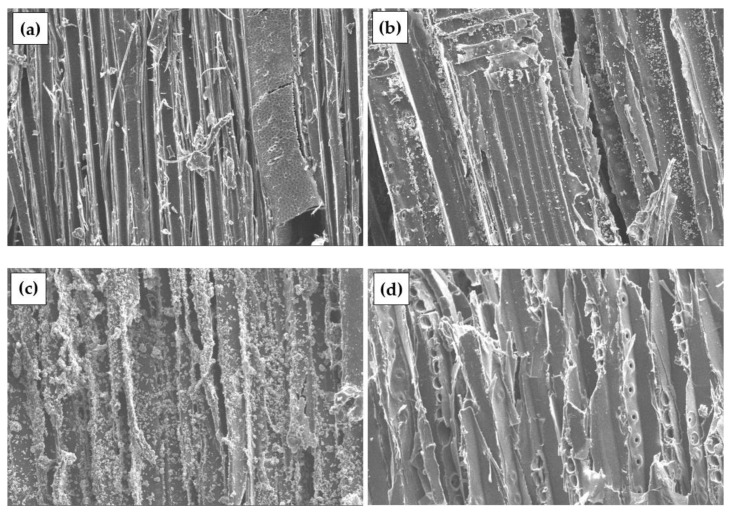
PLY particles with different surface preparation, ×500: (**a**) untreated; (**b**) S-treated; (**c**) C-treated; (**d**) P-treated.

Figure 6 shows untreated and chemically treated surfaces of PLY particles. Small amounts of fine particles are observed in chemically untreated waste. The effect of the chemical treatment is almost the same, but a higher amount of crystal formation is observed.

### 3.4. Structure of Engineered Wood

**Figure 7:**
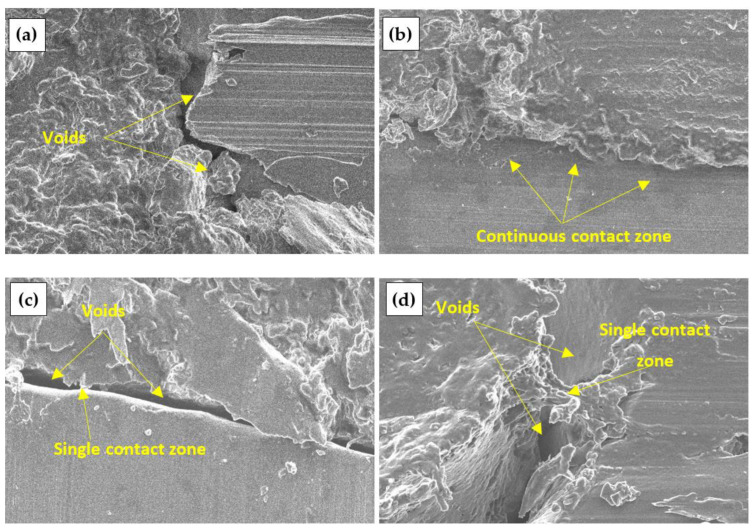
Engineered wood made from DW particles, ×5000: (**a**) untreated; (**b**) S-treated; (**c**) C-treated; (**d**) P-treated.

Figure 7 shows a view of engineered wood specimens made from untreated and chemically treated DW. When untreated and C- and P-treated DW is used, many voids between the particles and the binder are observed. Meanwhile, a homogeneous structure of the engineered wood specimens is observed in the engineered wood prepared from S-treated DW particles, i.e., without voids.

Figure 7a shows the contact zone between the binder and the aggregate of the engineered wood specimen made from untreated DW can be seen. The Figure 7a shows the sharp edges of the particles without formed contact areas. In Figure 7b,c, and d, the contact zones of engineered wood specimens prepared from chemically treated DW particles are noted. The formation of the best contact zone is observed when DW particles are treated with S.

**Figure 8:**
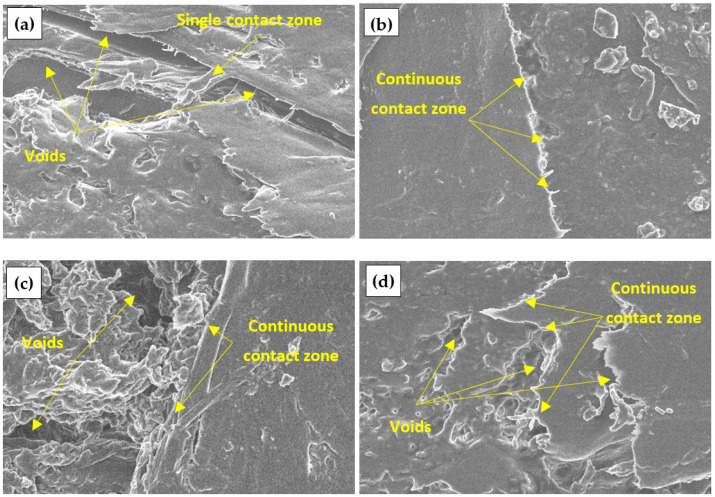
Engineered wood made from W particles, ×8000: (**a**) untreated; (**b**) S-treated; (**c**) C-treated; (**d**) P-treated.

A similar view can be seen in Figure 8 for engineered wood specimens made of W particles. A homogeneous structure without voids is observed in the specimens prepared from S-treated W particles (Figure 8b). The surface of the specimen is completely covered with the biopolyurethane binder, which results in the highest amount of contact zones. Voids are observed on the surfaces of specimens prepared from C and P-treated W particles, thus affecting the formation of contact zones.

**Figure 9:**
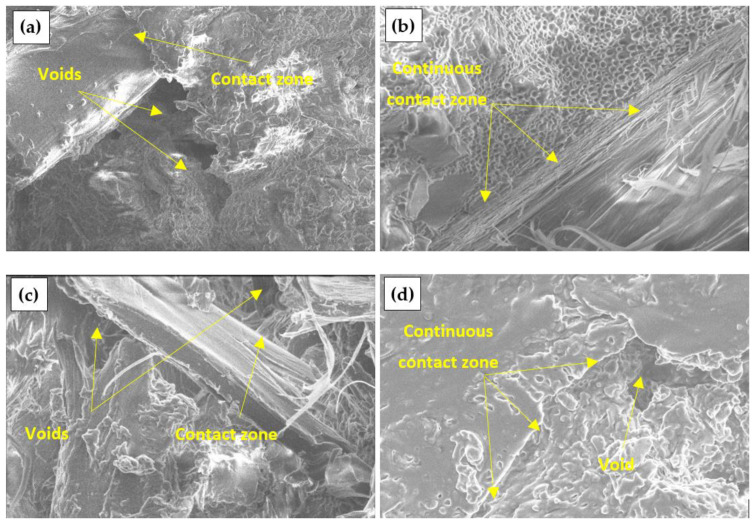
Engineered wood made from PLY particles, ×6500: (**a**) untreated; (**b**) S-treated; (**c**) C-treated; (**d**) P-treated.

Figure 9 shows engineered wood specimens made of PLY with a similar view. In this case, voids are observed on all surfaces of the specimens. As mentioned earlier, the binding material used in primary products, which then became waste materials, is likely to be partially or entirely removed during chemical treatment, resulting in the formation of additional voids.

Structural analysis shows that the values of compressive strength and tensile strength can be further increased. The greatest attention must be paid to the preparation of the wood waste surfaces, the improvement of the contact zones between the binding material and the wood particles, and the elimination of voids between the particles.

## 4. Conclusions

1. The chemical treatment of different wood wastes has different effects on the bulk density of the particles. The bulk density of chemically treated PLY particles decreased in all cases; the most significant decrease was 26.9%. In the case of DW, the bulk density increases under P and C treatments, i.e., the highest increase reaches 14.2%.
2. The granulometric composition of different wood wastes differs. In DW particles, 40% to 60% consist of the small fraction varying from 0 to 1.25 mm, and in PLY particles, the large fraction varying from 2.5 to 10 mm prevails and constitutes 72.5–88.6%.
3. The strength characteristics of wood waste-based engineered wood logs depend on the chemical treatment of the wood waste and the origin of the waste itself. The best effect is found when the W particles are treated with S. In this case, the compressive strength increases by 87%, and the tensile strength increases by 52.5%. According to the average values of both parameters, the obtained engineered wood with S-treated W particles can be assigned to T12 class based on the requirements of EN 338 [63] for sawn construction softwood.
4. Analysis of the structure shows that the chemical treatment of wood waste removes small particles from the surface of larger particles and increases their surface roughness.
5. Analysis of the structure of engineered wood samples shows that the use of untreated or improperly treated waste forms voids between the particles and does not create a reliable contact zone between the biopolyurethane binder and the wood waste particles.
6. The results of these studies underscore the need for further research and development in the field of engineered wood production. It is clear that the method of chemical or other treatment should be selected for each type of wood waste separately. Special attention should be paid to the waste wood with binders used in primary products, which at the end of their life, became waste.

In summary, the findings of this study have significant implications for the production of engineered wood products. The use of biopolyurethane as a binding material opens up the possibility to produce engineered wood products of large dimensions without thermal treatment and replace natural wood in many cases. However, further research is needed to evaluate durability and various strength parameters, as well as to explore possible reinforcement methods to achieve bending strength close to that of natural wood.

## Figures and Tables

**Table 1:** Granulometric composition of components in wt.%.

Indicators	Residues on Different Sizes Sieves, wt.%					
	0–0.315 mm	>0.315–0.63 mm	>0.63–1.25 mm	>1.25–2.5 mm	>2.5–5 mm	>5 mm
DW-Av./Std.	1.18/0.063	13.01/0.085	26.73/0.095	3.89/0.120	41.96/0.090	13.23/0.450
DWS-Av./Std.	3.70/0.115	19.12/0.296	32.87/0.376	4.70/0.138	35.58/0.187	4.03/1.091
DWC-Av./Std.	3.36/0.072	21.01/0.114	36.97/0.241	3.36/0.118	33.61/0.151	1.69/0.691
DWP-Av./Std.	3.13/0.095	19.53/0.154	36.72/0.383	4.69/0.195	34.38/0.263	1.55/0.902
PLY	1.18/0.027	8.12/0.070	14.89/0.230	3.38/0.110	49.41/0.121	23.02/0.265
PLYS	1.35/0.085	8.78/0.075	14.19/0.100	2.70/0.0800	47.97/0.090	25.01/0.229
PLYC	1.33/0.082	7.33/0.066	11.33/0.106	2.00/0.118	46.67/0.336	31.34/0.125
PLYP	2.01/0.111	8.72/0.147	12.75/0.160	2.01/0.131	47.65/0.286	26.86/0.806
W	0.51/0.026	3.70/0.361	8.25/0.265	1.85/0.218	51.01/0.965	34.68/1.133
WS	0/0	2.68/0.131	7.37/0.150	1.34/0.053	49.95/0.145	38.66/0.287
WC	0/0	3.33/0.046	8/0.167	1.33/0.050	55.33/0.669	32.01/0.469
WP	0.67/0.104	4.70/0.190	8.72/0.121	1.34/0.072	51.01/1.012	33.56/1.005

Note: Av.—average value, Std.—standard deviation. Average values are calculated based on three measurements.

## Data Availability

The original contributions presented in the study are included in the article, further inquiries can be directed to the corresponding author.
